# Properties and Activity of Peptide Derivatives of ACE2 Cellular Receptor and Their Interaction with SARS-CoV-2 S Protein Receptor-Binding Domain

**DOI:** 10.1134/S1607672922060126

**Published:** 2022-12-29

**Authors:** M. V. Sidorova, R. S. Bibilashvili, D. V. Avdeev, U. S. Kozhokar, M. E. Palkeeva, M. V. Ovchinnikov, A. S. Molokoedov, D. A. Shirokov, A. V. Semyonova, V. I. Uvarova, P. O. Kulyaev, E. V. Khvatov, A. A. Ignatova, A. V. Feofanov, D. I. Osolodkin, Yu. B. Porozov, L. I. Kozlovskaya, A. A. Ishmukhametov, Ye. V. Parfyonova, A. M. Egorov

**Affiliations:** 1 Chazov National Medical Research Center for Cardiology, Ministry of Health of the Russian Federation, Moscow, Russia; 2 Federal Research and Clinical Center of Physical Chemical Medicine, Federal Medical Biological Agency, Moscow, Russia; 3 Skryabin Moscow State Academy of Veterinary Medicine and Biotechnology, Moscow, Russia; 4 Chumakov Federal Scientific Center for Research and Development of Immune and Biological Products of the Russian Academy of Sciences (Institute of Poliomyelitis), Moscow, Russia; 5 Sirius University of Science and Technology, Sochi, Russia; 6 Institute of Bioorganic Chemistry, Russian Academy of Sciences, Moscow, Russia; 7 Lomonosov Moscow State University, Moscow, Russia; 8 Sechenov First Moscow State Medical University (Sechenov University), Moscow, Russia

**Keywords:** SARS-CoV-2, peptide inhibitors, RBD, ACE2, helicity, dissociation constant

## Abstract

The aim of this work was to design and characterize peptides based on the α-helices h1 and h2 of the ACE2 receptor, forming the interaction interface between the receptor-binding domain (RBD) of the SARS-CoV-2 S protein and the cellular ACE2 receptor. Monomeric and heterodimeric peptides connected by disulfide bonds at different positions were synthesized. Solubility, RBD-binding affinity, and peptide helicity were experimentally measured, and molecular dynamics simulation was performed in various solvents. It was established that the preservation of the helical conformation is a necessary condition for the binding of peptides to RBD. The peptides have a low degree of helicity and low affinity for RBD in water. Dimeric peptides have a higher degree of helicity than monomeric ones, probably due to the mutual influence of helices. The degree of helicity of the peptides in trifluoroethanol is the highest; however, for in vitro studies, the most suitable solvent is a water-ethanol mixture.

The COVID-19 pandemic and the emergence of new variants of its etiological agent, the SARS-CoV-2 virus, make studies on the search for effective tools for prevention and treatment of this infection relevant. At the initial stages of the interaction of the virus with the cell, the key role is played by the S protein, which forms spikes on the surface of the viral particle. The receptor-binding domain (RBD) of the S protein interacts with the interface of the cellular ACE2 receptor, which consists of h1 and h2 α-helices, and triggers the fusion of the cell and viral membranes for the penetration of the viral genome into the cytoplasm [1]. To date, to inhibit this interaction, monoclonal antibodies with neutralizing activity have been developed and used in clinical practice [2–4], which will later become a reference drug for new compounds. One of the approaches to design of the molecules capable of competitively preventing the binding of RBD of SARS-CoV-2 and related viruses to ACE2 is the design of peptides that mimic the structure and properties of the cellular ACE2 receptor. In 2020–2022, a significant number of peptide molecules based on the ACE2 sequence and aimed at interacting with the S protein [5] have been studied in silico, and only for some of them the effectiveness of inhibition of the formation of the RBD-ACE2 complex and (or) antiviral activity have been studied in vitro. Moreover, a few of synthesized compounds that exhibited a high (nanomolar) binding affinity in silico [6] and in vitro [7, 8] exhibited antiviral activity in cell or animal models [9–12].

To increase the binding efficiency, it is necessary to optimize the peptide structure in order to create a molecule that most closely resembles the ACE2 structure and provides a high-affinity interaction with RBD [13–15]. We have developed and synthesized monomeric peptides based on the amino acid sequence of antiparallel h1 and h2 ACE2 α-helices with modified residues. Monomeric peptides were linked by disulfide bridges at different positions to obtain heterodimeric “chimeric” peptides X1–4 (Table 1; synthesis of peptides X1, X2, and their monomers was described earlier [16]). For these peptides, experimental studies of solubility, binding affinity with RBD, and the degree of helicity, as well as molecular dynamics simulations in various solvents, were performed.

**Table 1.** Structure and properties of the studied peptides

Code, comment	Sequence	α-Helicity, %^а^			Kd for binding to RBD, µM^b^	
		H_2_O	WEM	TFE	H_2_O	WEM
h1 helix						
**MTI-23** (SPB1, control)	IEEQAKTFLDKFNHEAEDLFYQS-NH2	6.0	44.4	79.8	NB	3.57 ± 1.78
**h1–D–Cys** (21–42) h1	**c**EEQAKTFLDKFNHEAEDLFYk	6.2	39.2	72.5	–	–
**h1–Mpa** (21–42) h1	IEEQAKTFLDK[Mpa]FNHQAEDLFYk	6.0	37.8	85.3	NB	1.07 ± 0.36
**200h1** (21–44) h1	IEEQAKTFLDKFNHEAEDLFYQCS	3.9	40.3	52.4	NB	3.10 ± 1.03
**900h1** (21–44) h1	IEEQAKTFLDEFNEEAEDLFYQCS	NS*	37.2	73.7	NB	3.45 ± 2.42
h2 helix						
**h2** (67–89) h2	DKWSAFLKEQSTIAQ-Nle-YPLQECI	8.0	45.5	62.0	40 ± 8	2.58 ± 1.11
**h2-Cys** (67-89) h2	DKWSAFLKECSTIAQIYPLQEI	6.9	38.2	70.2	NB	3.87 ± 1.94
**900h2** (64–87) h2	NCGDKWSAFLKEQSTLAQ-Nle-YPLQE	7.2	51.6	65.2	1.7 ± 0.4	7.22 ± 3.61
Dimeric peptides						
**X1** (h1–D–Cys–h2)		8.0	41.1	95.2	4.2 ± 0.5	7.39 ± 5.54
**X2** (h1–Mpa–h2–Cys)		NS	51.7	82.5	12 ± 5	0.929 ± 0.371
**X3** (200h1 - 900h2) Cys43(h1)–Cys65(h2)		NS	55.0	98.0	NB	–
**X4** (900h1 - 900h2)		8.8	62.8	100.0	NB	0.882 ± 0.353

NB—no binding in the concentration range studied;

NS—not soluble;

“–”—not tested;

a—according to CD spectroscopy data;

b—according to thermophoresis data.

Affinity was studied by microthermophoresis [17], and the degree of α-helicity was studied by circular dichroism (CD) spectroscopy [18] (Table 1). The synthesized peptides are characterized by low water solubility. In aqueous solution, only two chimeric peptides (Kd X1: 4 μM, X2: 12 μM) and two monomeric peptides based on the h2 helix showed affinity for RBD (dissociation constants Kd were 40 μM for h2 and 2 μM for 900h2). It was found by the CD method that the peptides in aqueous solution have a low degree of helicity, which may be the cause for their low affinity for RBD.

Stabilization of secondary structure elements in peptides and proteins (in particular, α-helices) is a complex multifactorial process involving several different mechanisms. The spiralization-inducing effects include a decrease in the entropy contribution of solvent molecules due to the distortion of the hydrogen bond network between them and the side chains of amino acid residues in the peptide.

To increase the degree of helicity of the synthesized peptides, we selected solvents that stabilize their α-helical conformation and are also suitable for biological experiments.

The most suitable solvent for maintaining a stable helical conformation of peptides is trifluoroe-thanol (TFE) [19]. The degree of peptide helicity in it was the highest (from 50 to 100%). The dimeric peptides X3 and X4 retained a completely helical conformation in TFE. This solvent can be used to characterize the overall spiralization ability of the peptide. However, TFE is toxic to cell cultures and is unsuitable for in vitro studies at high concentrations.

The CD spectroscopy confirmed a higher degree of helicity of peptides in a water–ethanol mixture (WEM) (37–63%) compared to water. In a water–alcohol mixture, monomeric peptides exhibit affinity for RBD with dissociation constants in the range of 1‒4 and 2–8 μM for peptides based on the h1 and h2 α-helix sequences, respectively. Combining monomeric peptides into heterodimers leads to an increase in affinity when connecting “middle to middle” or “tail to head” (dissociation constants of dipeptides X2 and X4 < 1 μM) but does not influence affinity when connecting “head to tail” (dipeptide dissociation constant X1 ~7 μM) (Table 1). In addition, dimeric peptides have a higher degree of helicity than monomeric ones, which may be due to the mutual stabilizing effect of h1 and h2 helices.

In a molecular dynamics simulation (100–300 ns) in water, peptides, especially monomeric ones, tend to destabilize the structure and lose their helical conformation (Fig. 1).

**Fig. 1.**
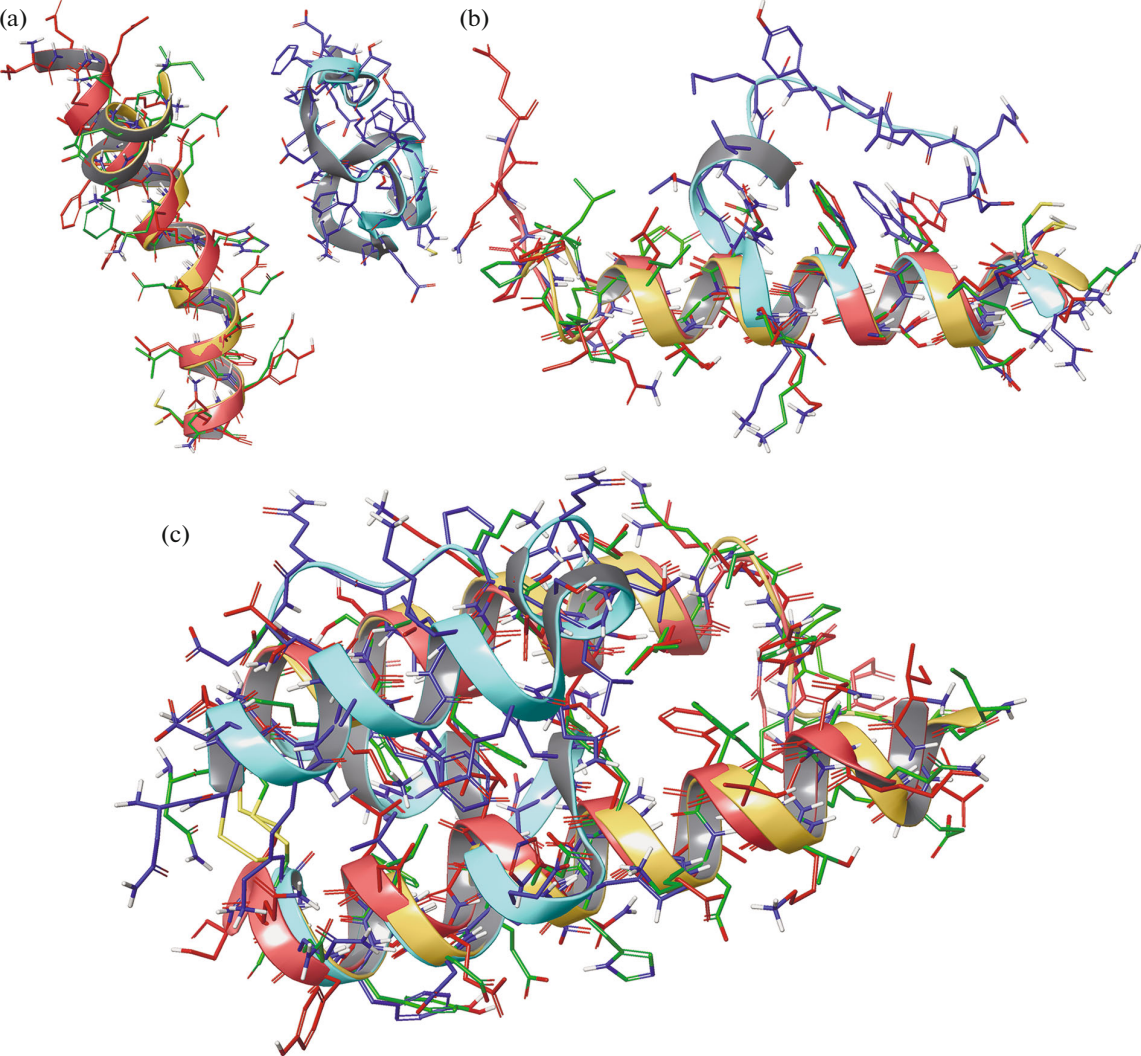
Representative structures of the monomeric peptides 200h1 (a) and 900h2 (b) and the chimeric peptide X3 derived from them (c) according to the results of molecular dynamics simulations. Structures in water, WEM, and TFE are colored in blue, red, and yellow, respectively, and superimposed by structural similarity. The superposition of the 200h1 structures is impossible due to the fundamental difference in the conformations.

The degree of helicity and stability of chimeric peptides in water is significantly higher than that of monomeric peptides, therefore supporting the hypothesis of mutual stabilization of helices in heterodimers. In a water–ethanol mixture and in TFE, the helical conformation remains fairly stable during the entire simulation time for both monomeric and chimeric peptides. This indicates the possibility of additional stabilization of the helical structure due to the properties of these solvents, which partially mimic the features of the protein environment of the helices in the receptor structure.

The proposed dimeric peptides based on the h1 and h2 helices of the ACE2 receptor connected by disulfide bridges, are capable of binding to the RBD of the SARS-CoV-2 S protein with an affinity in the submicromolar range, which exceeds the affinity of individual monomers. The formation of a helical structure of peptides increases the efficiency of binding to SARS-CoV-2 RBD: the affinity of peptides to RBD depends on the degree of α-helicity of their secondary structure, and the preservation of the original helical structure inherent in the ACE2 receptor is necessary for successful binding of the SARS-CoV-2 S-protein to RBD. The water–ethanol mixture maintains the helicity of the peptides and can be used for in vitro studies. Further stabilization of the peptide structure and selection of the optimal amino acid sequence and solvent should make it possible to design candidate molecules for studying the interaction between SARS-CoV-2 and the receptor in vivo.
